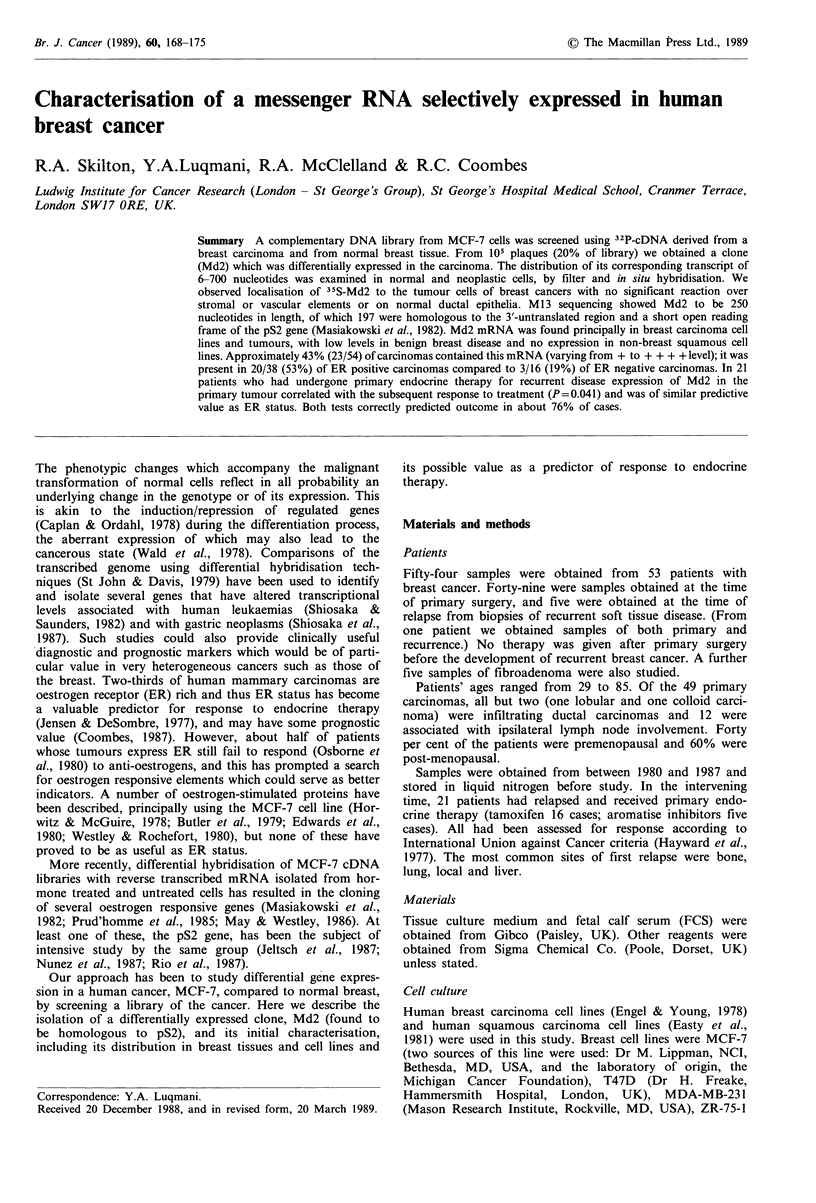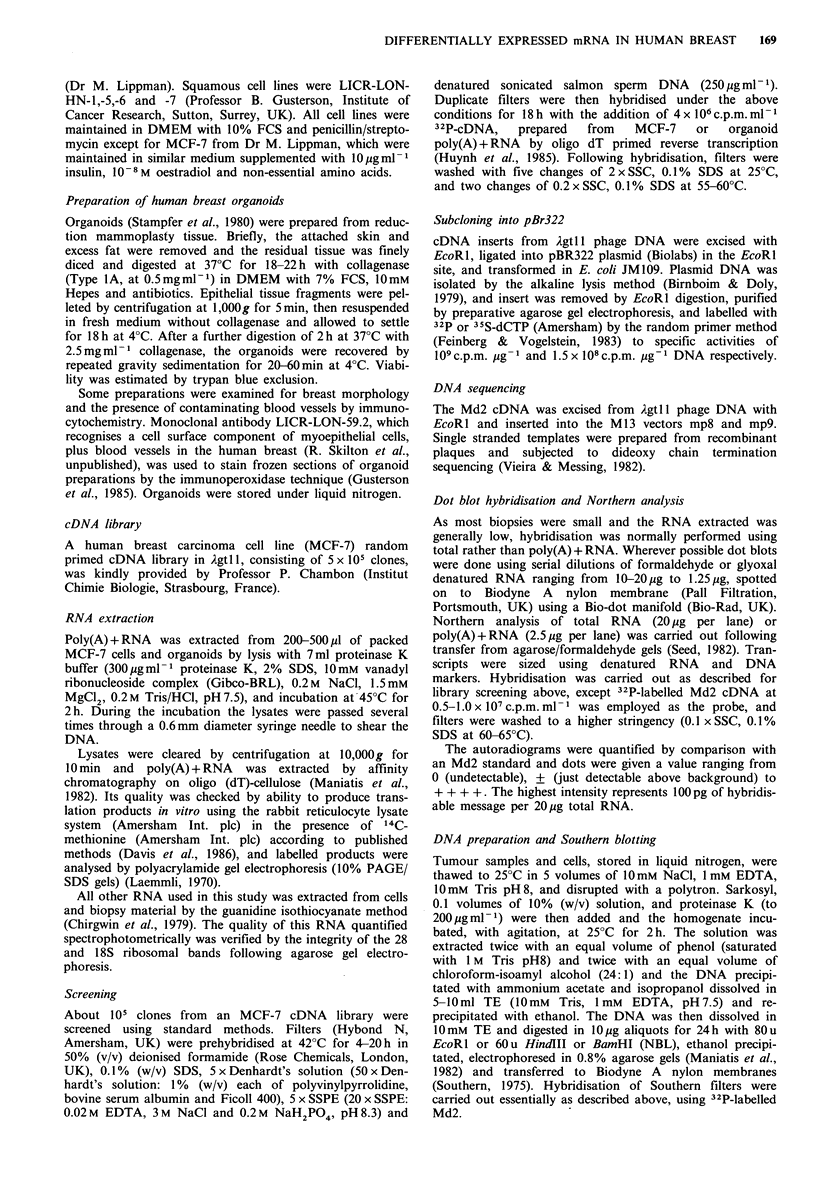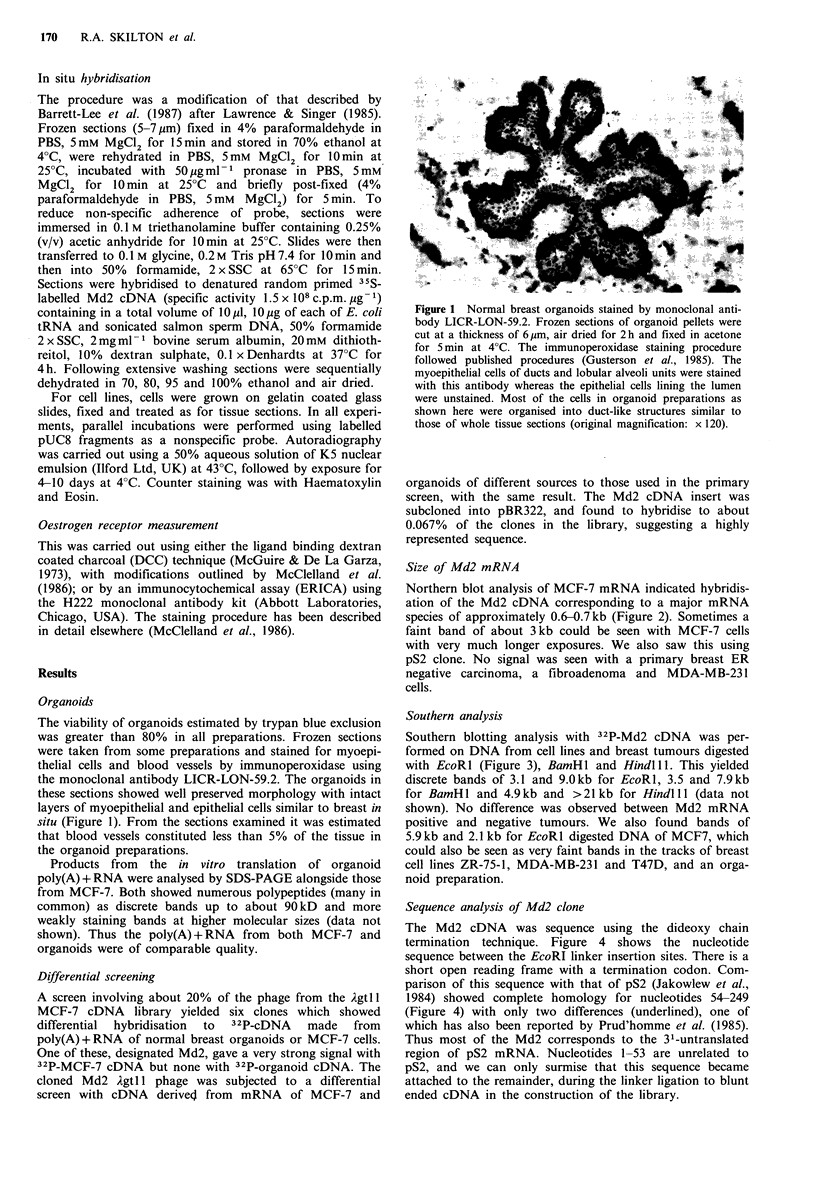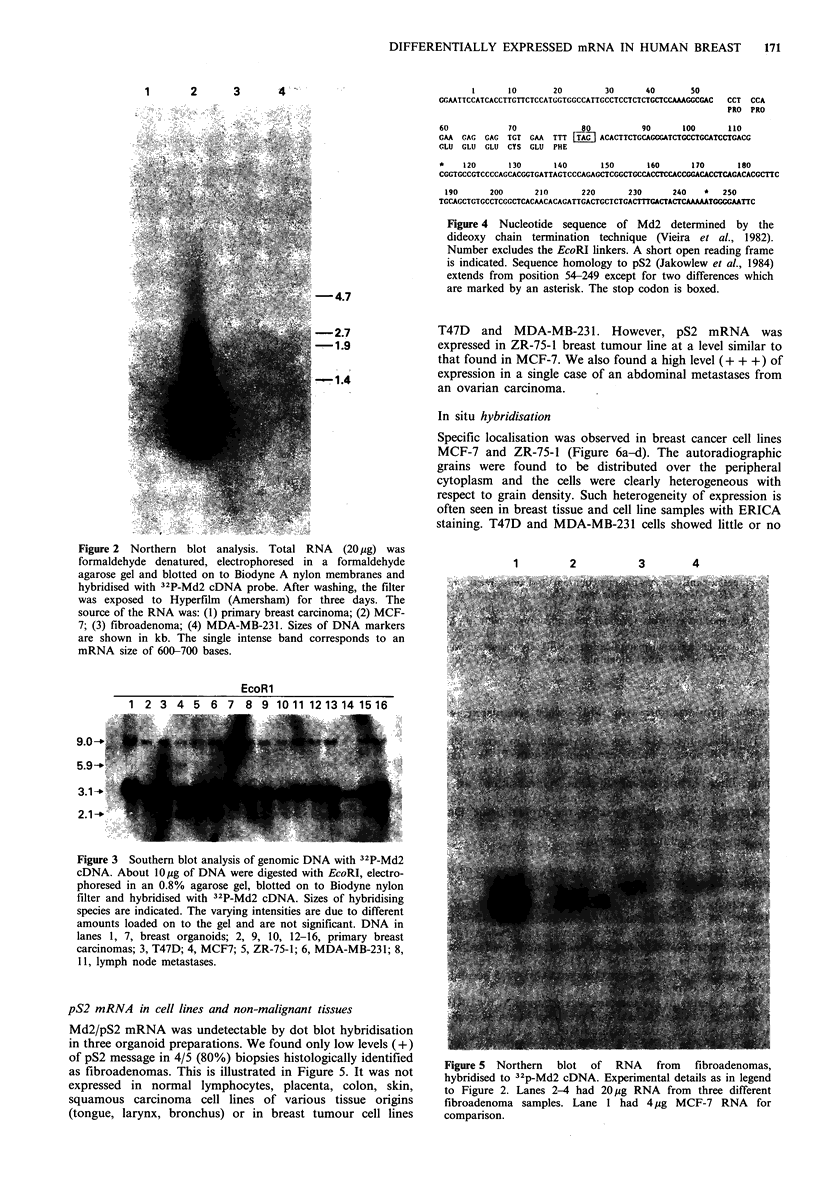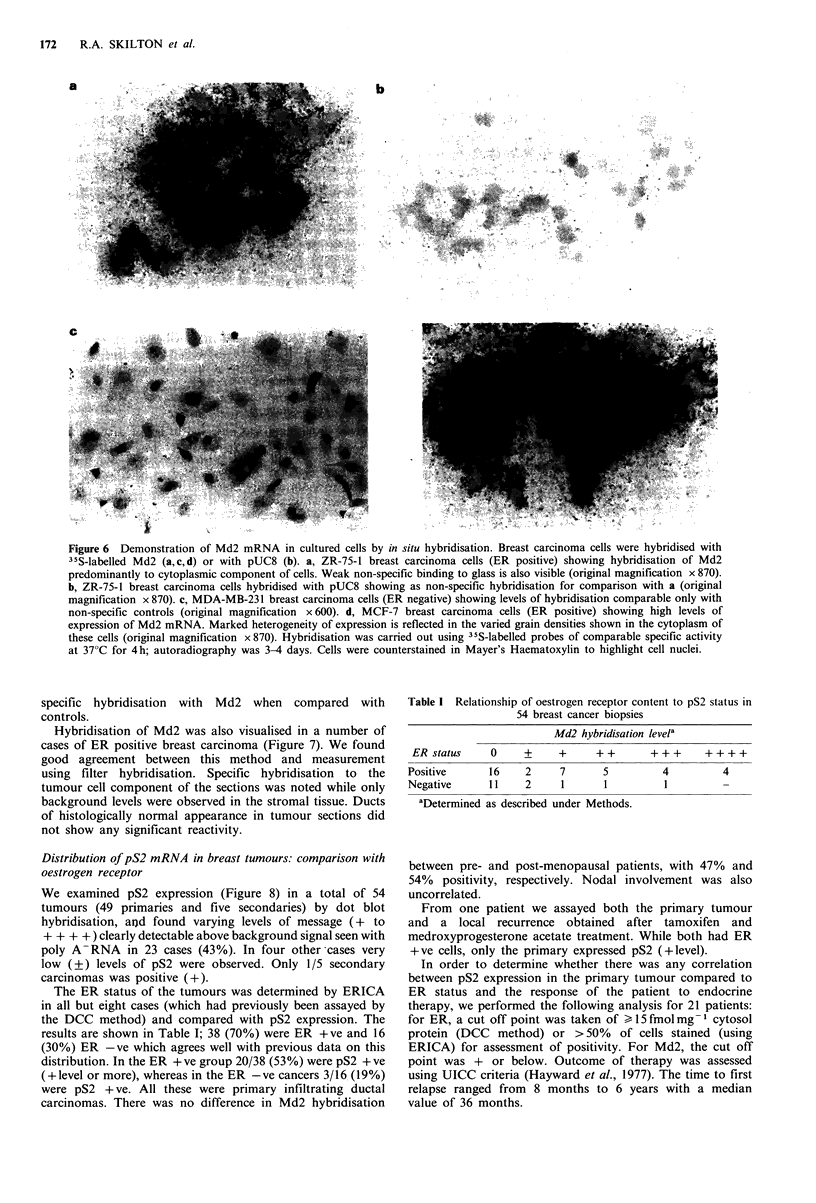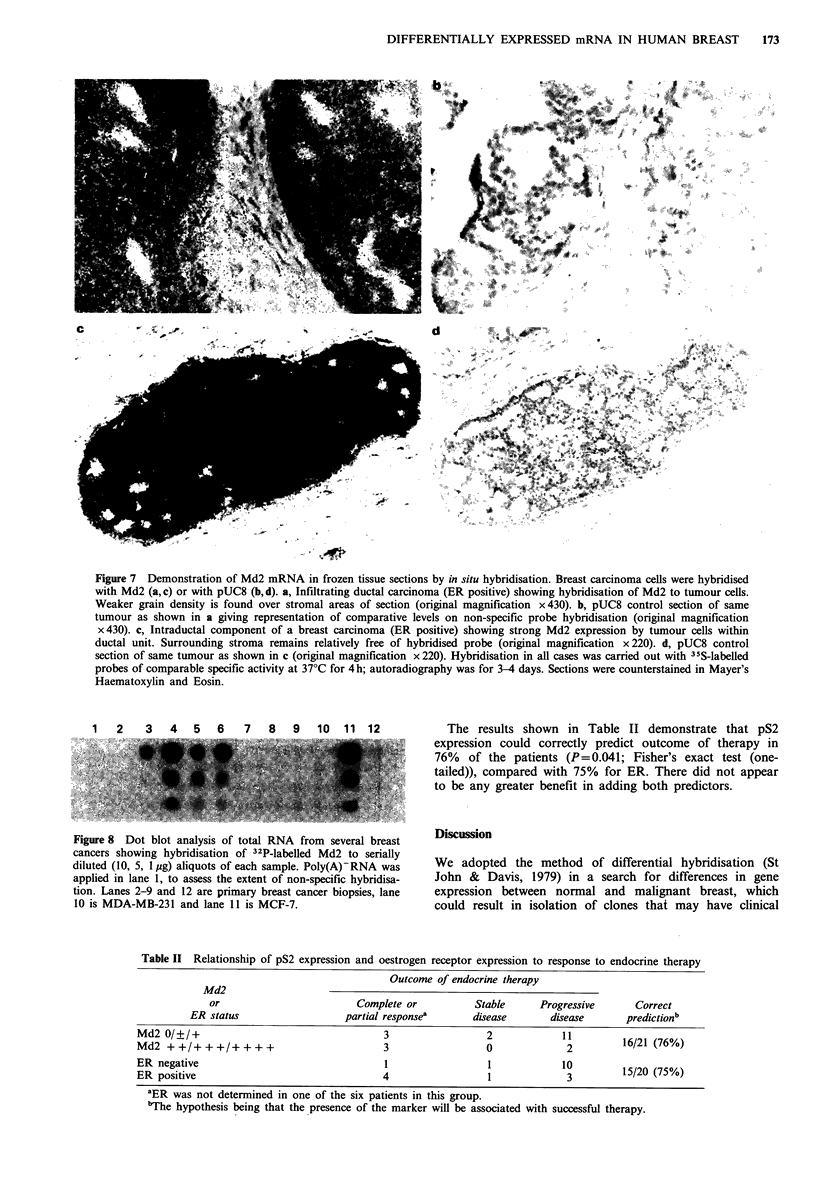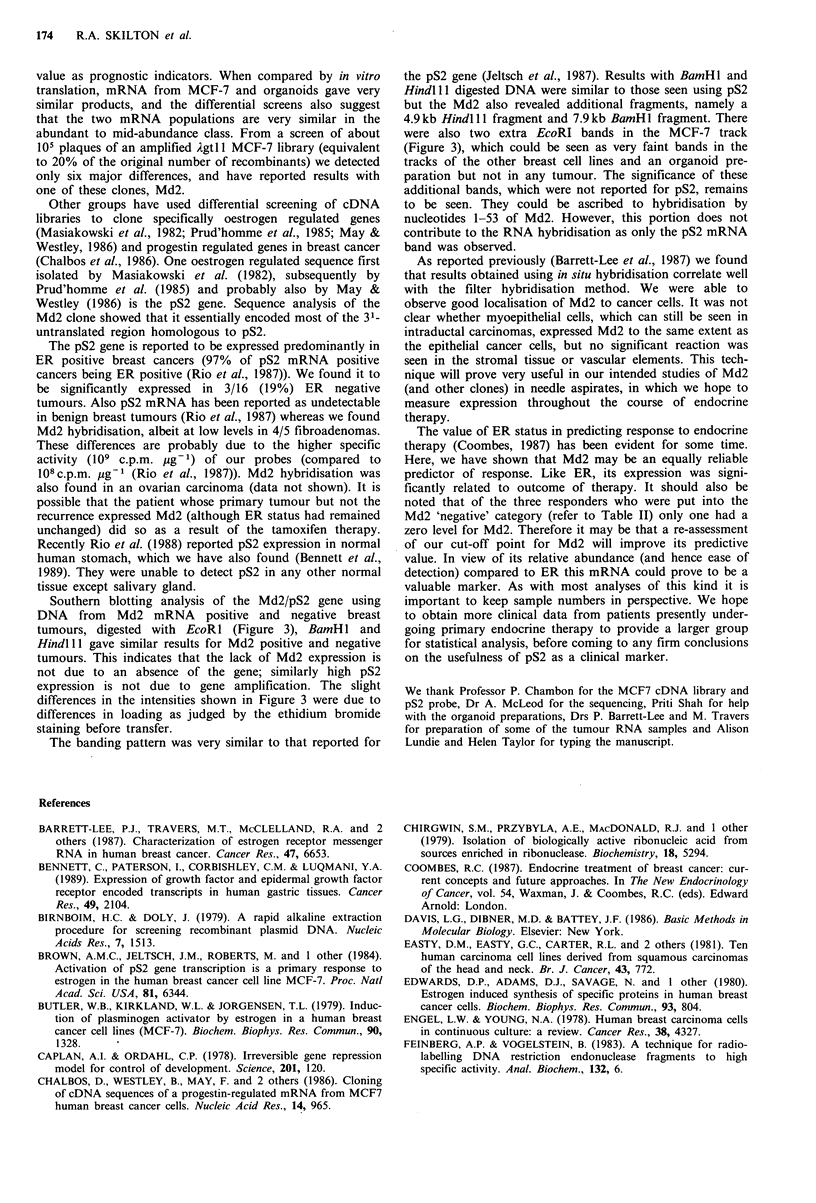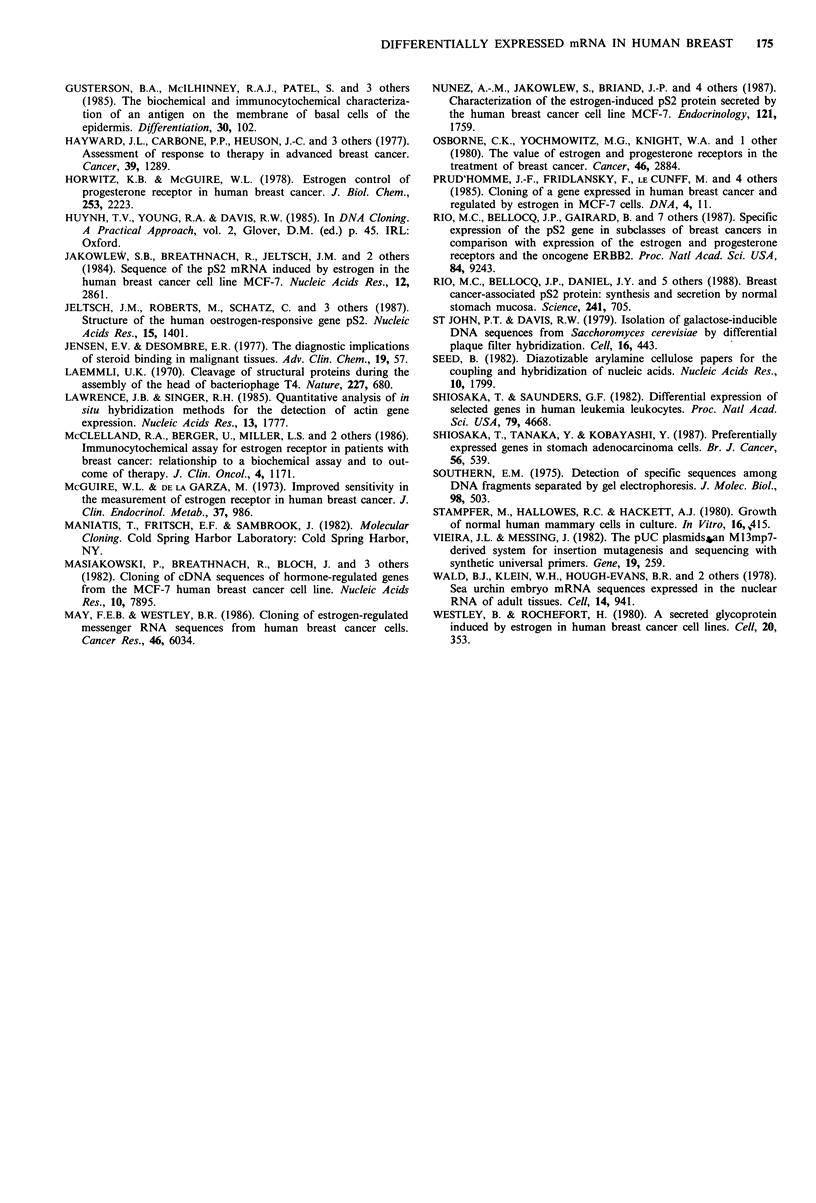# Characterisation of a messenger RNA selectively expressed in human breast cancer.

**DOI:** 10.1038/bjc.1989.245

**Published:** 1989-08

**Authors:** R. A. Skilton, Y. A. Luqmani, R. A. McClelland, R. C. Coombes

**Affiliations:** Ludwig Institute for Cancer Research, St George's Hospital Medical School, London, UK.

## Abstract

**Images:**


Br. J. Cancer (1989), 60, 168–175 © The Macmillan Press Ltd., 1989

### Characterisation of a messenger RNA selectively expressed in human breast cancer

R.A. Skilton, Y.A.Luqmani, R.A. McClelland & R.C. Coombes

*Ludwig Institute for Cancer Research (London – St George's Group), St George's Hospital Medical School, Cranmer Terrace, London SW17 0RE, UK.*

**Summary** A complementary DNA library from MCF-7 cells was screened using $^{32}$P-cDNA derived from a breast carcinoma and from normal breast tissue. From $10^5$ plaques (20% of library) we obtained a clone (Md2) which was differentially expressed in the carcinoma. The distribution of its corresponding transcript of 6–700 nucleotides was examined in normal and neoplastic cells, by filter and *in situ* hybridisation. We observed localisation of $^{35}$S-Md2 to the tumour cells of breast cancers with no significant reaction over stromal or vascular elements or on normal ductal epithelia. M13 sequencing showed Md2 to be 250 nucleotides in length, of which 197 were homologous to the 3′-untranslated region and a short open reading frame of the pS2 gene (Masiakowski *et al.*, 1982). Md2 mRNA was found principally in breast carcinoma cell lines and tumours, with low levels in benign breast disease and no expression in non-breast squamous cell lines. Approximately 43% (23/54) of carcinomas contained this mRNA (varying from + to + + + + level); it was present in 20/38 (53%) of ER positive carcinomas compared to 3/16 (19%) of ER negative carcinomas. In 21 patients who had undergone primary endocrine therapy for recurrent disease expression of Md2 in the primary tumour correlated with the subsequent response to treatment ($P$=0.041) and was of similar predictive value as ER status. Both tests correctly predicted outcome in about 76% of cases.

The phenotypic changes which accompany the malignant transformation of normal cells reflect in all probability an underlying change in the genotype or of its expression. This is akin to the induction/repression of regulated genes (Caplan & Ordahl, 1978) during the differentiation process, the aberrant expression of which may also lead to the cancerous state (Wald *et al.*, 1978). Comparisons of the transcribed genome using differential hybridisation techniques (St John & Davis, 1979) have been used to identify and isolate several genes that have altered transcriptional levels associated with human leukaemias (Shiosaka & Saunders, 1982) and with gastric neoplasms (Shiosaka *et al.*, 1987). Such studies could also provide clinically useful diagnostic and prognostic markers which would be of particular value in very heterogeneous cancers such as those of the breast. Two-thirds of human mammary carcinomas are oestrogen receptor (ER) rich and thus ER status has become a valuable predictor for response to endocrine therapy (Jensen & DeSombre, 1977), and may have some prognostic value (Coombes, 1987). However, about half of patients whose tumours express ER still fail to respond (Osborne *et al.*, 1980) to anti-oestrogens, and this has prompted a search for oestrogen responsive elements which could serve as better indicators. A number of oestrogen-stimulated proteins have been described, principally using the MCF-7 cell line (Horwitz & McGuire, 1978; Butler *et al.*, 1979; Edwards *et al.*, 1980; Westley & Rochefort, 1980), but none of these have proved to be as useful as ER status.

More recently, differential hybridisation of MCF-7 cDNA libraries with reverse transcribed mRNA isolated from hormone treated and untreated cells has resulted in the cloning of several oestrogen responsive genes (Masiakowski *et al.*, 1982; Prud'homme *et al.*, 1985; May & Westley, 1986). At least one of these, the pS2 gene, has been the subject of intensive study by the same group (Jeltsch *et al.*, 1987; Nunez *et al.*, 1987; Rio *et al.*, 1987).

Our approach has been to study differential gene expression in a human cancer, MCF-7, compared to normal breast, by screening a library of the cancer. Here we describe the isolation of a differentially expressed clone, Md2 (found to be homologous to pS2), and its initial characterisation, including its distribution in breast tissues and cell lines and its possible value as a predictor of response to endocrine therapy.

Correspondence: Y.A. Luqmani.
Received 20 December 1988, and in revised form, 20 March 1989.

### Materials and methods

### *Patients*

Fifty-four samples were obtained from 53 patients with breast cancer. Forty-nine were samples obtained at the time of primary surgery, and five were obtained at the time of relapse from biopsies of recurrent soft tissue disease. (From one patient we obtained samples of both primary and recurrence.) No therapy was given after primary surgery before the development of recurrent breast cancer. A further five samples of fibroadenoma were also studied.

Patients' ages ranged from 29 to 85. Of the 49 primary carcinomas, all but two (one lobular and one colloid carcinoma) were infiltrating ductal carcinomas and 12 were associated with ipsilateral lymph node involvement. Forty per cent of the patients were premenopausal and 60% were post-menopausal.

Samples were obtained from between 1980 and 1987 and stored in liquid nitrogen before study. In the intervening time, 21 patients had relapsed and received primary endocrine therapy (tamoxifen 16 cases; aromatise inhibitors five cases). All had been assessed for response according to International Union against Cancer criteria (Hayward *et al.*, 1977). The most common sites of first relapse were bone, lung, local and liver.

### *Materials*

Tissue culture medium and fetal calf serum (FCS) were obtained from Gibco (Paisley, UK). Other reagents were obtained from Sigma Chemical Co. (Poole, Dorset, UK) unless stated.

### *Cell culture*

Human breast carcinoma cell lines (Engel & Young, 1978) and human squamous carcinoma cell lines (Easty *et al.*, 1981) were used in this study. Breast cell lines were MCF-7 (two sources of this line were used: Dr M. Lippman, NCI, Bethesda, MD, USA, and the laboratory of origin, the Michigan Cancer Foundation), T47D (Dr H. Freake, Hammersmith Hospital, London, UK), MDA-MB-231 (Mason Research Institute, Rockville, MD, USA), ZR-75-1

(Dr M. Lippman). Squamous cell lines were LICR-LON-HN-1,-5,-6 and -7 (Professor B. Gusterson, Institute of Cancer Research, Sutton, Surrey, UK). All cell lines were maintained in DMEM with 10% FCS and penicillin/streptomycin except for MCF-7 from Dr M. Lippman, which were maintained in similar medium supplemented with $10\,\mu g\,ml^{-1}$ insulin, $10^{-8}$ M oestradiol and non-essential amino acids.

*Preparation of human breast organoids*

Organoids (Stampfer *et al.*, 1980) were prepared from reduction mammoplasty tissue. Briefly, the attached skin and excess fat were removed and the residual tissue was finely diced and digested at 37°C for 18–22 h with collagenase (Type 1A, at $0.5\,mg\,ml^{-1}$) in DMEM with 7% FCS, 10 mM Hepes and antibiotics. Epithelial tissue fragments were pelleted by centrifugation at 1,000 *g* for 5 min, then resuspended in fresh medium without collagenase and allowed to settle for 18 h at 4°C. After a further digestion of 2 h at 37°C with $2.5\,mg\,ml^{-1}$ collagenase, the organoids were recovered by repeated gravity sedimentation for 20–60 min at 4°C. Viability was estimated by trypan blue exclusion.

Some preparations were examined for breast morphology and the presence of contaminating blood vessels by immunocytochemistry. Monoclonal antibody LICR-LON-59.2, which recognises a cell surface component of myoepithelial cells, plus blood vessels in the human breast (R. Skilton *et al.*, unpublished), was used to stain frozen sections of organoid preparations by the immunoperoxidase technique (Gusterson *et al.*, 1985). Organoids were stored under liquid nitrogen.

*cDNA library*

A human breast carcinoma cell line (MCF-7) random primed cDNA library in λgt11, consisting of $5 \times 10^5$ clones, was kindly provided by Professor P. Chambon (Institut Chimie Biologie, Strasbourg, France).

*RNA extraction*

Poly(A)+RNA was extracted from 200–500 μl of packed MCF-7 cells and organoids by lysis with 7 ml proteinase K buffer ($300\,\mu g\,ml^{-1}$ proteinase K, 2% SDS, 10 mM vanadyl ribonucleoside complex (Gibco-BRL), 0.2 M NaCl, 1.5 mM $MgCl_2$, 0.2 M Tris/HCl, pH 7.5), and incubation at 45°C for 2 h. During the incubation the lysates were passed several times through a 0.6 mm diameter syringe needle to shear the DNA.

Lysates were cleared by centrifugation at 10,000 *g* for 10 min and poly(A)+RNA was extracted by affinity chromatography on oligo (dT)-cellulose (Maniatis *et al.*, 1982). Its quality was checked by ability to produce translation products *in vitro* using the rabbit reticulocyte lysate system (Amersham Int. plc) in the presence of $^{14}C$-methionine (Amersham Int. plc) according to published methods (Davis *et al.*, 1986), and labelled products were analysed by polyacrylamide gel electrophoresis (10% PAGE/SDS gels) (Laemmli, 1970).

All other RNA used in this study was extracted from cells and biopsy material by the guanidine isothiocyanate method (Chirgwin *et al.*, 1979). The quality of this RNA quantified spectrophotometrically was verified by the integrity of the 28 and 18S ribosomal bands following agarose gel electrophoresis.

*Screening*

About $10^5$ clones from an MCF-7 cDNA library were screened using standard methods. Filters (Hybond N, Amersham, UK) were prehybridised at 42°C for 4–20 h in 50% (v/v) deionised formamide (Rose Chemicals, London, UK), 0.1% (w/v) SDS, 5 × Denhardt's solution (50 × Denhardt's solution: 1% (w/v) each of polyvinylpyrrolidine, bovine serum albumin and Ficoll 400), 5 × SSPE (20 × SSPE: 0.02 M EDTA, 3 M NaCl and 0.2 M $NaH_2PO_4$, pH 8.3) and denatured sonicated salmon sperm DNA ($250\,\mu g\,ml^{-1}$). Duplicate filters were then hybridised under the above conditions for 18 h with the addition of $4 \times 10^6$ c.p.m. $ml^{-1}$ $^{32}P$-cDNA, prepared from MCF-7 or organoid poly(A)+RNA by oligo dT primed reverse transcription (Huynh *et al.*, 1985). Following hybridisation, filters were washed with five changes of 2 × SSC, 0.1% SDS at 25°C, and two changes of 0.2 × SSC, 0.1% SDS at 55–60°C.

*Subcloning into pBr322*

cDNA inserts from λgt11 phage DNA were excised with *Eco*R1, ligated into pBR322 plasmid (Biolabs) in the *Eco*R1 site, and transformed in *E. coli* JM109. Plasmid DNA was isolated by the alkaline lysis method (Birnboim & Doly, 1979), and insert was removed by *Eco*R1 digestion, purified by preparative agarose gel electrophoresis, and labelled with $^{32}P$ or $^{35}S$-dCTP (Amersham) by the random primer method (Feinberg & Vogelstein, 1983) to specific activities of $10^9$ c.p.m. $\mu g^{-1}$ and $1.5 \times 10^8$ c.p.m. $\mu g^{-1}$ DNA respectively.

*DNA sequencing*

The Md2 cDNA was excised from λgt11 phage DNA with *Eco*R1 and inserted into the M13 vectors mp8 and mp9. Single stranded templates were prepared from recombinant plaques and subjected to dideoxy chain termination sequencing (Vieira & Messing, 1982).

*Dot blot hybridisation and Northern analysis*

As most biopsies were small and the RNA extracted was generally low, hybridisation was normally performed using total rather than poly(A)+RNA. Wherever possible dot blots were done using serial dilutions of formaldehyde or glyoxal denatured RNA ranging from 10–20 μg to 1.25 μg, spotted on to Biodyne A nylon membrane (Pall Filtration, Portsmouth, UK) using a Bio-dot manifold (Bio-Rad, UK). Northern analysis of total RNA (20 μg per lane) or poly(A)+RNA (2.5 μg per lane) was carried out following transfer from agarose/formaldehyde gels (Seed, 1982). Transcripts were sized using denatured RNA and DNA markers. Hybridisation was carried out as described for library screening above, except $^{32}P$-labelled Md2 cDNA at $0.5–1.0 \times 10^7$ c.p.m. $ml^{-1}$ was employed as the probe, and filters were washed to a higher stringency (0.1 × SSC, 0.1% SDS at 60–65°C).

The autoradiograms were quantified by comparison with an Md2 standard and dots were given a value ranging from 0 (undetectable), ± (just detectable above background) to + + + +. The highest intensity represents 100 pg of hybridisable message per 20 μg total RNA.

*DNA preparation and Southern blotting*

Tumour samples and cells, stored in liquid nitrogen, were thawed to 25°C in 5 volumes of 10 mM NaCl, 1 mM EDTA, 10 mM Tris pH 8, and disrupted with a polytron. Sarkosyl, 0.1 volumes of 10% (w/v) solution, and proteinase K (to $200\,\mu g\,ml^{-1}$) were then added and the homogenate incubated, with agitation, at 25°C for 2 h. The solution was extracted twice with an equal volume of phenol (saturated with 1 M Tris pH8) and twice with an equal volume of chloroform-isoamyl alcohol (24:1) and the DNA precipitated with ammonium acetate and isopropanol dissolved in 5–10 ml TE (10 mM Tris, 1 mM EDTA, pH 7.5) and reprecipitated with ethanol. The DNA was then dissolved in 10 mM TE and digested in 10 μg aliquots for 24 h with 80 u *Eco*R1 or 60 u *Hind*III or *Bam*HI (NBL), ethanol precipitated, electrophoresed in 0.8% agarose gels (Maniatis *et al.*, 1982) and transferred to Biodyne A nylon membranes (Southern, 1975). Hybridisation of Southern filters were carried out essentially as described above, using $^{32}P$-labelled Md2.

*In situ hybridisation*

The procedure was a modification of that described by Barrett-Lee *et al.* (1987) after Lawrence & Singer (1985). Frozen sections (5–7 μm) fixed in 4% paraformaldehyde in PBS, 5 mM $MgCl_2$ for 15 min and stored in 70% ethanol at 4°C, were rehydrated in PBS, 5 mM $MgCl_2$ for 10 min at 25°C, incubated with 50 $\mu g\,ml^{-1}$ pronase in PBS, 5 mM $MgCl_2$ for 10 min at 25°C and briefly post-fixed (4% paraformaldehyde in PBS, 5 mM $MgCl_2$) for 5 min. To reduce non-specific adherence of probe, sections were immersed in 0.1 M triethanolamine buffer containing 0.25% (v/v) acetic anhydride for 10 min at 25°C. Slides were then transferred to 0.1 M glycine, 0.2 M Tris pH 7.4 for 10 min and then into 50% formamide, 2 × SSC at 65°C for 15 min. Sections were hybridised to denatured random primed $^{35}S$-labelled Md2 cDNA (specific activity $1.5 \times 10^8$ c.p.m. $\mu g^{-1}$) containing in a total volume of 10 μl, 10 μg of each of *E. coli* tRNA and sonicated salmon sperm DNA, 50% formamide 2 × SSC, 2 mg $ml^{-1}$ bovine serum albumin, 20 mM dithiothreitol, 10% dextran sulphate, 0.1 × Denhardts at 37°C for 4 h. Following extensive washing sections were sequentially dehydrated in 70, 80, 95 and 100% ethanol and air dried.

For cell lines, cells were grown on gelatin coated glass slides, fixed and treated as for tissue sections. In all experiments, parallel incubations were performed using labelled pUC8 fragments as a nonspecific probe. Autoradiography was carried out using a 50% aqueous solution of K5 nuclear emulsion (Ilford Ltd, UK) at 43°C, followed by exposure for 4–10 days at 4°C. Counter staining was with Haematoxylin and Eosin.

*Oestrogen receptor measurement*

This was carried out using either the ligand binding dextran coated charcoal (DCC) technique (McGuire & De La Garza, 1973), with modifications outlined by McClelland *et al.* (1986); or by an immunocytochemical assay (ERICA) using the H222 monoclonal antibody kit (Abbott Laboratories, Chicago, USA). The staining procedure has been described in detail elsewhere (McClelland *et al.*, 1986).

### Results

*Organoids*

The viability of organoids estimated by trypan blue exclusion was greater than 80% in all preparations. Frozen sections were taken from some preparations and stained for myoepithelial cells and blood vessels by immunoperoxidase using the monoclonal antibody LICR-LON-59.2. The organoids in these sections showed well preserved morphology with intact layers of myoepithelial and epithelial cells similar to breast *in situ* (Figure 1). From the sections examined it was estimated that blood vessels constituted less than 5% of the tissue in the organoid preparations.

Products from the *in vitro* translation of organoid poly(A) + RNA were analysed by SDS-PAGE alongside those from MCF-7. Both showed numerous polypeptides (many in common) as discrete bands up to about 90 kD and more weakly staining bands at higher molecular sizes (data not shown). Thus the poly(A) + RNA from both MCF-7 and organoids were of comparable quality.

*Differential screening*

A screen involving about 20% of the phage from the λgt11 MCF-7 cDNA library yielded six clones which showed differential hybridisation to $^{32}P$-cDNA made from poly(A) + RNA of normal breast organoids or MCF-7 cells. One of these, designated Md2, gave a very strong signal with $^{32}P$-MCF-7 cDNA but none with $^{32}P$-organoid cDNA. The cloned Md2 λgt11 phage was subjected to a differential screen with cDNA derived from mRNA of MCF-7 and organoids of different sources to those used in the primary screen, with the same result. The Md2 cDNA insert was subcloned into pBR322, and found to hybridise to about 0.067% of the clones in the library, suggesting a highly represented sequence.

**Figure 1** Normal breast organoids stained by monoclonal antibody LICR-LON-59.2. Frozen sections of organoid pellets were cut at a thickness of 6 μm, air dried for 2 h and fixed in acetone for 5 min at 4°C. The immunoperoxidase staining procedure followed published procedures (Gusterson *et al.*, 1985). The myoepithelial cells of ducts and lobular alveoli units were stained with this antibody whereas the epithelial cells lining the lumen were unstained. Most of the cells in organoid preparations as shown here were organised into duct-like structures similar to those of whole tissue sections (original magnification: × 120).

*Size of Md2 mRNA*

Northern blot analysis of MCF-7 mRNA indicated hybridisation of the Md2 cDNA corresponding to a major mRNA species of approximately 0.6–0.7 kb (Figure 2). Sometimes a faint band of about 3 kb could be seen with MCF-7 cells with very much longer exposures. We also saw this using pS2 clone. No signal was seen with a primary breast ER negative carcinoma, a fibroadenoma and MDA-MB-231 cells.

*Southern analysis*

Southern blotting analysis with $^{32}P$-Md2 cDNA was performed on DNA from cell lines and breast tumours digested with *Eco*R1 (Figure 3), *Bam*H1 and *Hind*111. This yielded discrete bands of 3.1 and 9.0 kb for *Eco*R1, 3.5 and 7.9 kb for *Bam*H1 and 4.9 kb and >21 kb for *Hind*111 (data not shown). No difference was observed between Md2 mRNA positive and negative tumours. We also found bands of 5.9 kb and 2.1 kb for *Eco*R1 digested DNA of MCF7, which could also be seen as very faint bands in the tracks of breast cell lines ZR-75-1, MDA-MB-231 and T47D, and an organoid preparation.

*Sequence analysis of Md2 clone*

The Md2 cDNA was sequence using the dideoxy chain termination technique. Figure 4 shows the nucleotide sequence between the *Eco*RI linker insertion sites. There is a short open reading frame with a termination codon. Comparison of this sequence with that of pS2 (Jakowlew *et al.*, 1984) showed complete homology for nucleotides 54–249 (Figure 4) with only two differences (underlined), one of which has also been reported by Prud'homme *et al.* (1985). Thus most of the Md2 corresponds to the 3[1]-untranslated region of pS2 mRNA. Nucleotides 1–53 are unrelated to pS2, and we can only surmise that this sequence became attached to the remainder, during the linker ligation to blunt ended cDNA in the construction of the library.

**Figure 2** Northern blot analysis. Total RNA (20 $\mu$g) was formaldehyde denatured, electrophoresed in a formaldehyde agarose gel and blotted on to Biodyne A nylon membranes and hybridised with $^{32}$P-Md2 cDNA probe. After washing, the filter was exposed to Hyperfilm (Amersham) for three days. The source of the RNA was: (1) primary breast carcinoma; (2) MCF-7; (3) fibroadenoma; (4) MDA-MB-231. Sizes of DNA markers are shown in kb. The single intense band corresponds to an mRNA size of 600–700 bases.

**Figure 3** Southern blot analysis of genomic DNA with $^{32}$P-Md2 cDNA. About 10 $\mu$g of DNA were digested with *Eco*RI, electrophoresed in an 0.8% agarose gel, blotted on to Biodyne nylon filter and hybridised with $^{32}$P-Md2 cDNA. Sizes of hybridising species are indicated. The varying intensities are due to different amounts loaded on to the gel and are not significant. DNA in lanes 1, 7, breast organoids; 2, 9, 10, 12–16, primary breast carcinomas; 3, T47D; 4, MCF7; 5, ZR-75-1; 6, MDA-MB-231; 8, 11, lymph node metastases.

### *pS2 mRNA in cell lines and non-malignant tissues*

Md2/pS2 mRNA was undetectable by dot blot hybridisation in three organoid preparations. We found only low levels (+) of pS2 message in 4/5 (80%) biopsies histologically identified as fibroadenomas. This is illustrated in Figure 5. It was not expressed in normal lymphocytes, placenta, colon, skin, squamous carcinoma cell lines of various tissue origins (tongue, larynx, bronchus) or in breast tumour cell lines T47D and MDA-MB-231. However, pS2 mRNA was expressed in ZR-75-1 breast tumour line at a level similar to that found in MCF-7. We also found a high level (+ + +) of expression in a single case of an abdominal metastases from an ovarian carcinoma.

```
       1        10         20          30         40         50
GGAATTCCATCACCTTGTTCTCCATGGTGGCCATTGCCTCCTCTCTGCTCCAAAGGCGAC    CCT   CCA
                                                                 PRO   PRO

60             70             80             90       100       110
GAA  GAG  GAG  TGT  GAA  TTT [TAG] ACACTTCTGCAGGGATCTGCCTGCATCCTGACG
GLU  GLU  GLU  CYS  GLU  PHE

*     120        130        140         150        160        170        180
CGGTGCCGTCCCCAGCACGGTGATTAGTCCCAGAGCTCGGCTGCCACCTCCACCGGACACCTCAGACACGCTTC

 190        200        210        220         230        240   *   250
TGCAGCTGTGCCTCGGCTCACAACACAGATTGACTGCTCTGACTTTGACTACTCAAAAATGGGGAATTC
```

**Figure 4** Nucleotide sequence of Md2 determined by the dideoxy chain termination technique (Vieira *et al.*, 1982). Number excludes the *Eco*RI linkers. A short open reading frame is indicated. Sequence homology to pS2 (Jakowlew *et al.*, 1984) extends from position 54–249 except for two differences which are marked by an asterisk. The stop codon is boxed.

### In situ *hybridisation*

Specific localisation was observed in breast cancer cell lines MCF-7 and ZR-75-1 (Figure 6a–d). The autoradiographic grains were found to be distributed over the peripheral cytoplasm and the cells were clearly heterogeneous with respect to grain density. Such heterogeneity of expression is often seen in breast tissue and cell line samples with ERICA staining. T47D and MDA-MB-231 cells showed little or no

**Figure 5** Northern blot of RNA from fibroadenomas, hybridised to $^{32}$p-Md2 cDNA. Experimental details as in legend to Figure 2. Lanes 2–4 had 20 $\mu$g RNA from three different fibroadenoma samples. Lane 1 had 4 $\mu$g MCF-7 RNA for comparison.

**Figure 6** Demonstration of Md2 mRNA in cultured cells by *in situ* hybridisation. Breast carcinoma cells were hybridised with $^{35}$S-labelled Md2 (**a**,**c**,**d**) or with pUC8 (**b**). **a**, ZR-75-1 breast carcinoma cells (ER positive) showing hybridisation of Md2 predominantly to cytoplasmic component of cells. Weak non-specific binding to glass is also visible (original magnification ×870). **b**, ZR-75-1 breast carcinoma cells hybridised with pUC8 showing as non-specific hybridisation for comparison with **a** (original magnification ×870). **c**, MDA-MB-231 breast carcinoma cells (ER negative) showing levels of hybridisation comparable only with non-specific controls (original magnification ×600). **d**, MCF-7 breast carcinoma cells (ER positive) showing high levels of expression of Md2 mRNA. Marked heterogeneity of expression is reflected in the varied grain densities shown in the cytoplasm of these cells (original magnification ×870). Hybridisation was carried out using $^{35}$S-labelled probes of comparable specific activity at 37°C for 4h; autoradiography was 3–4 days. Cells were counterstained in Mayer's Haematoxylin to highlight cell nuclei.

specific hybridisation with Md2 when compared with controls.

Hybridisation of Md2 was also visualised in a number of cases of ER positive breast carcinoma (Figure 7). We found good agreement between this method and measurement using filter hybridisation. Specific hybridisation to the tumour cell component of the sections was noted while only background levels were observed in the stromal tissue. Ducts of histologically normal appearance in tumour sections did not show any significant reactivity.

*Distribution of pS2 mRNA in breast tumours: comparison with oestrogen receptor*

We examined pS2 expression (Figure 8) in a total of 54 tumours (49 primaries and five secondaries) by dot blot hybridisation, and found varying levels of message (+ to ++++) clearly detectable above background signal seen with poly A$^-$RNA in 23 cases (43%). In four other cases very low (±) levels of pS2 were observed. Only 1/5 secondary carcinomas was positive (+).

The ER status of the tumours was determined by ERICA in all but eight cases (which had previously been assayed by the DCC method) and compared with pS2 expression. The results are shown in Table I; 38 (70%) were ER +ve and 16 (30%) ER −ve which agrees well with previous data on this distribution. In the ER +ve group 20/38 (53%) were pS2 +ve (+level or more), whereas in the ER −ve cancers 3/16 (19%) were pS2 +ve. All these were primary infiltrating ductal carcinomas. There was no difference in Md2 hybridisation

**Table I** Relationship of oestrogen receptor content to pS2 status in 54 breast cancer biopsies

| | *Md2 hybridisation level*[a] | | | | | |
|---|---|---|---|---|---|---|
| *ER status* | 0 | ± | + | ++ | +++ | ++++ |
| Positive | 16 | 2 | 7 | 5 | 4 | 4 |
| Negative | 11 | 2 | 1 | 1 | 1 | – |

[a]Determined as described under Methods.

between pre- and post-menopausal patients, with 47% and 54% positivity, respectively. Nodal involvement was also uncorrelated.

From one patient we assayed both the primary tumour and a local recurrence obtained after tamoxifen and medroxyprogesterone acetate treatment. While both had ER +ve cells, only the primary expressed pS2 (+level).

In order to determine whether there was any correlation between pS2 expression in the primary tumour compared to ER status and the response of the patient to endocrine therapy, we performed the following analysis for 21 patients: for ER, a cut off point was taken of $\geqslant 15\,\mathrm{fmol\,mg^{-1}}$ cytosol protein (DCC method) or >50% of cells stained (using ERICA) for assessment of positivity. For Md2, the cut off point was + or below. Outcome of therapy was assessed using UICC criteria (Hayward *et al.*, 1977). The time to first relapse ranged from 8 months to 6 years with a median value of 36 months.

**Figure 7** Demonstration of Md2 mRNA in frozen tissue sections by *in situ* hybridisation. Breast carcinoma cells were hybridised with Md2 (**a**, **c**) or with pUC8 (**b**, **d**). **a**, Infiltrating ductal carcinoma (ER positive) showing hybridisation of Md2 to tumour cells. Weaker grain density is found over stromal areas of section (original magnification ×430). **b**, pUC8 control section of same tumour as shown in **a** giving representation of comparative levels on non-specific probe hybridisation (original magnification ×430). **c**, Intraductal component of a breast carcinoma (ER positive) showing strong Md2 expression by tumour cells within ductal unit. Surrounding stroma remains relatively free of hybridised probe (original magnification ×220). **d**, pUC8 control section of same tumour as shown in **c** (original magnification ×220). Hybridisation in all cases was carried out with $^{35}$S-labelled probes of comparable specific activity at 37°C for 4 h; autoradiography was for 3–4 days. Sections were counterstained in Mayer's Haematoxylin and Eosin.

**Figure 8** Dot blot analysis of total RNA from several breast cancers showing hybridisation of $^{32}$P-labelled Md2 to serially diluted (10, 5, 1 μg) aliquots of each sample. Poly(A)$^-$RNA was applied in lane 1, to assess the extent of non-specific hybridisation. Lanes 2–9 and 12 are primary breast cancer biopsies, lane 10 is MDA-MB-231 and lane 11 is MCF-7.

The results shown in Table II demonstrate that pS2 expression could correctly predict outcome of therapy in 76% of the patients ($P=0.041$; Fisher's exact test (one-tailed)), compared with 75% for ER. There did not appear to be any greater benefit in adding both predictors.

### Discussion

We adopted the method of differential hybridisation (St John & Davis, 1979) in a search for differences in gene expression between normal and malignant breast, which could result in isolation of clones that may have clinical

**Table II** Relationship of pS2 expression and oestrogen receptor expression to response to endocrine therapy

| *Md2 or ER status* | *Outcome of endocrine therapy* | | | |
|---|---|---|---|---|
| | *Complete or partial response*[a] | *Stable disease* | *Progressive disease* | *Correct prediction*[b] |
| Md2 0/±/+ | 3 | 2 | 11 | 16/21 (76%) |
| Md2 ++/+++/++++ | 3 | 0 | 2 | |
| ER negative | 1 | 1 | 10 | 15/20 (75%) |
| ER positive | 4 | 1 | 3 | |

[a]ER was not determined in one of the six patients in this group.
[b]The hypothesis being that the presence of the marker will be associated with successful therapy.

value as prognostic indicators. When compared by *in vitro* translation, mRNA from MCF-7 and organoids gave very similar products, and the differential screens also suggest that the two mRNA populations are very similar in the abundant to mid-abundance class. From a screen of about $10^5$ plaques of an amplified λgt11 MCF-7 library (equivalent to 20% of the original number of recombinants) we detected only six major differences, and have reported results with one of these clones, Md2.

Other groups have used differential screening of cDNA libraries to clone specifically oestrogen regulated genes (Masiakowski *et al.*, 1982; Prud'homme *et al.*, 1985; May & Westley, 1986) and progestin regulated genes in breast cancer (Chalbos *et al.*, 1986). One oestrogen regulated sequence first isolated by Masiakowski *et al.* (1982), subsequently by Prud'homme *et al.* (1985) and probably also by May & Westley (1986) is the pS2 gene. Sequence analysis of the Md2 clone showed that it essentially encoded most of the 3¹-untranslated region homologous to pS2.

The pS2 gene is reported to be expressed predominantly in ER positive breast cancers (97% of pS2 mRNA positive cancers being ER positive (Rio *et al.*, 1987)). We found it to be significantly expressed in 3/16 (19%) ER negative tumours. Also pS2 mRNA has been reported as undetectable in benign breast tumours (Rio *et al.*, 1987) whereas we found Md2 hybridisation, albeit at low levels in 4/5 fibroadenomas. These differences are probably due to the higher specific activity ($10^9$ c.p.m. $\mu g^{-1}$) of our probes (compared to $10^8$ c.p.m. $\mu g^{-1}$ (Rio *et al.*, 1987)). Md2 hybridisation was also found in an ovarian carcinoma (data not shown). It is possible that the patient whose primary tumour but not the recurrence expressed Md2 (although ER status had remained unchanged) did so as a result of the tamoxifen therapy. Recently Rio *et al.* (1988) reported pS2 expression in normal human stomach, which we have also found (Bennett *et al.*, 1989). They were unable to detect pS2 in any other normal tissue except salivary gland.

Southern blotting analysis of the Md2/pS2 gene using DNA from Md2 mRNA positive and negative breast tumours, digested with *Eco*R1 (Figure 3), *Bam*H1 and *Hind*111 gave similar results for Md2 positive and negative tumours. This indicates that the lack of Md2 expression is not due to an absence of the gene; similarly high pS2 expression is not due to gene amplification. The slight differences in the intensities shown in Figure 3 were due to differences in loading as judged by the ethidium bromide staining before transfer.

The banding pattern was very similar to that reported for the pS2 gene (Jeltsch *et al.*, 1987). Results with *Bam*H1 and *Hind*111 digested DNA were similar to those seen using pS2 but the Md2 also revealed additional fragments, namely a 4.9 kb *Hind*111 fragment and 7.9 kb *Bam*H1 fragment. There were also two extra *Eco*RI bands in the MCF-7 track (Figure 3), which could be seen as very faint bands in the tracks of the other breast cell lines and an organoid preparation but not in any tumour. The significance of these additional bands, which were not reported for pS2, remains to be seen. They could be ascribed to hybridisation by nucleotides 1–53 of Md2. However, this portion does not contribute to the RNA hybridisation as only the pS2 mRNA band was observed.

As reported previously (Barrett-Lee *et al.*, 1987) we found that results obtained using *in situ* hybridisation correlate well with the filter hybridisation method. We were able to observe good localisation of Md2 to cancer cells. It was not clear whether myoepithelial cells, which can still be seen in intraductal carcinomas, expressed Md2 to the same extent as the epithelial cancer cells, but no significant reaction was seen in the stromal tissue or vascular elements. This technique will prove very useful in our intended studies of Md2 (and other clones) in needle aspirates, in which we hope to measure expression throughout the course of endocrine therapy.

The value of ER status in predicting response to endocrine therapy (Coombes, 1987) has been evident for some time. Here, we have shown that Md2 may be an equally reliable predictor of response. Like ER, its expression was significantly related to outcome of therapy. It should also be noted that of the three responders who were put into the Md2 'negative' category (refer to Table II) only one had a zero level for Md2. Therefore it may be that a re-assessment of our cut-off point for Md2 will improve its predictive value. In view of its relative abundance (and hence ease of detection) compared to ER this mRNA could prove to be a valuable marker. As with most analyses of this kind it is important to keep sample numbers in perspective. We hope to obtain more clinical data from patients presently undergoing primary endocrine therapy to provide a larger group for statistical analysis, before coming to any firm conclusions on the usefulness of pS2 as a clinical marker.

We thank Professor P. Chambon for the MCF7 cDNA library and pS2 probe, Dr A. McLeod for the sequencing, Priti Shah for help with the organoid preparations, Drs P. Barrett-Lee and M. Travers for preparation of some of the tumour RNA samples and Alison Lundie and Helen Taylor for typing the manuscript.

### References

BARRETT-LEE, P.J., TRAVERS, M.T., McCLELLAND, R.A. and 2 others (1987). Characterization of estrogen receptor messenger RNA in human breast cancer. *Cancer Res.*, **47**, 6653.

BENNETT, C., PATERSON, I., CORBISHLEY, C.M. & LUQMANI, Y.A. (1989). Expression of growth factor and epidermal growth factor receptor encoded transcripts in human gastric tissues. *Cancer Res.*, **49**, 2104.

BIRNBOIM, H.C. & DOLY, J. (1979). A rapid alkaline extraction procedure for screening recombinant plasmid DNA. *Nucleic Acids Res.*, **7**, 1513.

BROWN, A.M.C., JELTSCH, J.M., ROBERTS, M. and 1 other (1984). Activation of pS2 gene transcription is a primary response to estrogen in the human breast cancer cell line MCF-7. *Proc. Natl Acad. Sci. USA*, **81**, 6344.

BUTLER, W.B., KIRKLAND, W.L. & JORGENSEN, T.L. (1979). Induction of plasminogen activator by estrogen in a human breast cancer cell lines (MCF-7). *Biochem. Biophys. Res. Commun.*, **90**, 1328.

CAPLAN, A.I. & ORDAHL, C.P. (1978). Irreversible gene repression model for control of development. *Science*, **201**, 120.

CHALBOS, D., WESTLEY, B., MAY, F. and 2 others (1986). Cloning of cDNA sequences of a progestin-regulated mRNA from MCF7 human breast cancer cells. *Nucleic Acid Res.*, **14**, 965.

CHIRGWIN, S.M., PRZYBYLA, A.E., MacDONALD, R.J. and 1 other (1979). Isolation of biologically active ribonucleic acid from sources enriched in ribonuclease. *Biochemistry*, **18**, 5294.

COOMBES, R.C. (1987). Endocrine treatment of breast cancer: current concepts and future approaches. In *The New Endocrinology of Cancer*, vol. 54, Waxman, J. & Coombes, R.C. (eds). Edward Arnold: London.

DAVIS, L.G., DIBNER, M.D. & BATTEY, J.F. (1986). *Basic Methods in Molecular Biology*. Elsevier: New York.

EASTY, D.M., EASTY, G.C., CARTER, R.L. and 2 others (1981). Ten human carcinoma cell lines derived from squamous carcinomas of the head and neck. *Br. J. Cancer*, **43**, 772.

EDWARDS, D.P., ADAMS, D.J., SAVAGE, N. and 1 other (1980). Estrogen induced synthesis of specific proteins in human breast cancer cells. *Biochem. Biophys. Res. Commun.*, **93**, 804.

ENGEL, L.W. & YOUNG, N.A. (1978). Human breast carcinoma cells in continuous culture: a review. *Cancer Res.*, **38**, 4327.

FEINBERG, A.P. & VOGELSTEIN, B. (1983). A technique for radiolabelling DNA restriction endonuclease fragments to high specific activity. *Anal. Biochem.*, **132**, 6.

GUSTERSON, B.A., McILHINNEY, R.A.J., PATEL, S. and 3 others (1985). The biochemical and immunocytochemical characterization of an antigen on the membrane of basal cells of the epidermis. *Differentiation*, **30**, 102.

HAYWARD, J.L., CARBONE, P.P., HEUSON, J.-C. and 3 others (1977). Assessment of response to therapy in advanced breast cancer. *Cancer*, **39**, 1289.

HORWITZ, K.B. & McGUIRE, W.L. (1978). Estrogen control of progesterone receptor in human breast cancer. *J. Biol. Chem.*, **253**, 2223.

HUYNH, T.V., YOUNG, R.A. & DAVIS, R.W. (1985). In *DNA Cloning. A Practical Approach*, vol. 2, Glover, D.M. (ed.) p. 45. IRL: Oxford.

JAKOWLEW, S.B., BREATHNACH, R., JELTSCH, J.M. and 2 others (1984). Sequence of the pS2 mRNA induced by estrogen in the human breast cancer cell line MCF-7. *Nucleic Acids Res.*, **12**, 2861.

JELTSCH, J.M., ROBERTS, M., SCHATZ, C. and 3 others (1987). Structure of the human oestrogen-responsive gene pS2. *Nucleic Acids Res.*, **15**, 1401.

JENSEN, E.V. & DESOMBRE, E.R. (1977). The diagnostic implications of steroid binding in malignant tissues. *Adv. Clin. Chem.*, **19**, 57.

LAEMMLI, U.K. (1970). Cleavage of structural proteins during the assembly of the head of bacteriophage T4. *Nature*, **227**, 680.

LAWRENCE, J.B. & SINGER, R.H. (1985). Quantitative analysis of *in situ* hybridization methods for the detection of actin gene expression. *Nucleic Acids Res.*, **13**, 1777.

McCLELLAND, R.A., BERGER, U., MILLER, L.S. and 2 others (1986). Immunocytochemical assay for estrogen receptor in patients with breast cancer: relationship to a biochemical assay and to outcome of therapy. *J. Clin. Oncol.*, **4**, 1171.

McGUIRE, W.L. & DE LA GARZA, M. (1973). Improved sensitivity in the measurement of estrogen receptor in human breast cancer. *J. Clin. Endocrinol. Metab.*, **37**, 986.

MANIATIS, T., FRITSCH, E.F. & SAMBROOK, J. (1982). *Molecular Cloning*. Cold Spring Harbor Laboratory: Cold Spring Harbor, NY.

MASIAKOWSKI, P., BREATHNACH, R., BLOCH, J. and 3 others (1982). Cloning of cDNA sequences of hormone-regulated genes from the MCF-7 human breast cancer cell line. *Nucleic Acids Res.*, **10**, 7895.

MAY, F.E.B. & WESTLEY, B.R. (1986). Cloning of estrogen-regulated messenger RNA sequences from human breast cancer cells. *Cancer Res.*, **46**, 6034.

NUNEZ, A.-M., JAKOWLEW, S., BRIAND, J.-P. and 4 others (1987). Characterization of the estrogen-induced pS2 protein secreted by the human breast cancer cell line MCF-7. *Endocrinology*, **121**, 1759.

OSBORNE, C.K., YOCHMOWITZ, M.G., KNIGHT, W.A. and 1 other (1980). The value of estrogen and progesterone receptors in the treatment of breast cancer. *Cancer*, **46**, 2884.

PRUD'HOMME, J.-F., FRIDLANSKY, F., LE CUNFF, M. and 4 others (1985). Cloning of a gene expressed in human breast cancer and regulated by estrogen in MCF-7 cells. *DNA*, **4**, 11.

RIO, M.C., BELLOCQ, J.P., GAIRARD, B. and 7 others (1987). Specific expression of the pS2 gene in subclasses of breast cancers in comparison with expression of the estrogen and progesterone receptors and the oncogene ERBB2. *Proc. Natl Acad. Sci. USA*, **84**, 9243.

RIO, M.C., BELLOCQ, J.P., DANIEL, J.Y. and 5 others (1988). Breast cancer-associated pS2 protein: synthesis and secretion by normal stomach mucosa. *Science*, **241**, 705.

ST JOHN, P.T. & DAVIS, R.W. (1979). Isolation of galactose-inducible DNA sequences from *Sacchoromyces cerevisiae* by differential plaque filter hybridization. *Cell*, **16**, 443.

SEED, B. (1982). Diazotizable arylamine cellulose papers for the coupling and hybridization of nucleic acids. *Nucleic Acids Res.*, **10**, 1799.

SHIOSAKA, T. & SAUNDERS, G.F. (1982). Differential expression of selected genes in human leukemia leukocytes. *Proc. Natl Acad. Sci. USA*, **79**, 4668.

SHIOSAKA, T., TANAKA, Y. & KOBAYASHI, Y. (1987). Preferentially expressed genes in stomach adenocarcinoma cells. *Br. J. Cancer*, **56**, 539.

SOUTHERN, E.M. (1975). Detection of specific sequences among DNA fragments separated by gel electrophoresis. *J. Molec. Biol.*, **98**, 503.

STAMPFER, M., HALLOWES, R.C. & HACKETT, A.J. (1980). Growth of normal human mammary cells in culture. *In Vitro*, **16**, 415.

VIEIRA, J.L. & MESSING, J. (1982). The pUC plasmids, an M13mp7-derived system for insertion mutagenesis and sequencing with synthetic universal primers. *Gene*, **19**, 259.

WALD, B.J., KLEIN, W.H., HOUGH-EVANS, B.R. and 2 others (1978). Sea urchin embryo mRNA sequences expressed in the nuclear RNA of adult tissues. *Cell*, **14**, 941.

WESTLEY, B. & ROCHEFORT, H. (1980). A secreted glycoprotein induced by estrogen in human breast cancer cell lines. *Cell*, **20**, 353.